# Competition in human genetic technologies: The current US legal landscape

**DOI:** 10.1016/j.ajhg.2025.12.012

**Published:** 2026-01-21

**Authors:** Abdulai I. Rashid, Nicole A. Rincon, Nathan Rihani, Jennifer K. Wagner

**Affiliations:** 1 Brooks Kushman, Royal Oak, MI 48067, USA; 2 Scura, Wigfield, Heyer, Stevens, & Cammarota, LLP, Secaucus, NJ 07094, USA; 3 School of Engineering Design and Innovation, Penn State University, University Park, PA 16802, USA; 4 Department of Anthropology, Penn State University, University Park, PA 16802, USA; 5 Department of Biomedical Engineering, Penn State University, University Park, PA 16802, USA; 6 Penn State Dickinson Law, University Park, PA 16802, USA; 7 Institute for Computational and Data Sciences, Penn State University, University Park, PA 16802, USA; 8 Huck Institute of the Life Sciences, Penn State University, University Park, PA 16802, USA; 9 Rock Ethics Institute, Penn State University, University Park, PA 16802, USA; 10 Social Science Research Institute, Penn State University, University Park, PA 16802, USA

**Keywords:** ELSI, innovation, consumer protection, antitrust law, Federal Trade Commission, human genetics

## Abstract

Competition plays a crucial role in driving innovation in the industry of human genetics and genomics technologies. However, the US’s policy on competition (such as enforcement of antitrust laws) has shifted over time, affecting the level of regulatory scrutiny that business decisions in the industry will receive. Here, we offer an overview of this changing legal landscape, noting key policy changes at the Federal Trade Commission (FTC) and Department of Justice (DOJ) relevant to the human genetics and genomics industry. Focusing on the regulatory challenge of Illumina’s acquisition of Grail as a case study, we highlight how shifting anti-competition enforcement policy could affect spin-offs, startups, and industry consolidations. Balancing competition and consumer protection policies remains essential for the continued advancement of human genetic and genomic technologies. We offer this perspective in the hopes that it will help inform scientists in the field of the relevant legal considerations and stimulate discussions to shape policy—public and private alike—in ways that promote responsible innovation in genetic and genomic science and technology.

## Teaser

Here, we offer a perspective on the US legal landscape regarding the promotion of competition, with specific attention given to the human genetics and genomics industry. We highlight current policy on mergers, non-competition agreements, and exclusive dealings and use Illumina and Grail as a case study.

## Introduction

Competition plays an important role in driving innovation in science—including human genetics and genomics—rousing increased investment in research and development^1^ and motivating scientists to work toward achieving major breakthroughs and to seek rapid advancements. Promoting the business of science is not, however, without its “dark side,”^2^ as intense or unbridled competition can stifle collaboration in many ways. For example, excessive competition could drive science underground, where methods and claims are not subjected to peer review and where data are instead stockpiled, guarded, and leveraged as strategic business weapons rather than preserved, curated, and championed for broad public benefit.

Additional business concerns in the human genetics industry are asset transfers. While the bankruptcy proceedings of 23andMe grabbed extensive public, expert, and policymaker attention in 2025, concerns about transfers of assets (which could include a wide array of valuable property, including but not limited to personal genomic and related data) through bankruptcies specifically and mergers and acquisitions more generally are not without precedent (see web resources). For example, concerns were voiced in 2009 when deCODE Genetics filed for Chapter 11 bankruptcy protections in the US, selling its business assets, restructuring, and ultimately being acquired by Amgen in 2012 in a swift all-cash deal without prior regulatory approval, which ultimately ended the direct-to-consumer business of deCODEme (see web resources). Another historical example in human genetics of oversight concerns over deals that could consolidate sizable market power occurred in 2012 with Roche’s attempted takeover of Illumina. While the takeover was ultimately abandoned following intense regulatory scrutiny, concerns focused on the concentration of market power related to core genetic technology and data (see web resources). While national security concerns about potential bulk transfers of Americans’ human genetics data have been amplified recently (e.g., with a 2024 executive order^3^ and subsequent final rule issued by the Department of Justice [DOJ],^4^ a notice issued by the Cybersecurity & Infrastructure Security Agency,^5^ and a policy issued by the National Institutes of Health^6^), even such cross-border regulatory concerns are not new. One need only recall the apprehensions voiced in 2012 when Complete Genomics was acquired by BGI-Shenzhen, causing unease about foreign control over genetic technologies and data, as well as calling into question the adequacy of regulatory frameworks for protection and use of sensitive human genetic data (see web resources).

Over the past quarter-century, the Federal Trade Commission (FTC) has shaped human genetics industry dynamics in ways often overlooked by ELSI scholars, whose attention has focused squarely on the oversight authority and related regulatory and enforcement activities of the US Food and Drug Administration (FDA). The FTC has played an important role in our increasingly complex datafied culture (characterized in the US by a weak data protection framework). The FTC’s activities in personal genomics, mobile health, and other consumer areas with emerging technologies are worth knowing too.^7^^,^^8^ Here, we offer a concise overview of key policy changes at the FTC specifically relevant to promoting competition in the human genetics industry, which we hope will inform geneticists and enable more efficient communications and productive decision-making among geneticists and business partners in their respective “C-suites” (i.e., chief financial officers, chief legal officers, and chief executive officers).

## Overview of the FTC’s competition authority and recent policy shifts

### Regulatory authority

A basic understanding of the relevant statutory authority given to the FTC is helpful for understanding the impacts of antitrust regulation on the human genetics industry (see Box 1 for assistance with legal terminology). The main federal antitrust statutes that govern mergers are the Sherman Act,^9^ the FTC Act,^10^ the Clayton Act of 1914,^11^ and the Hart-Scott-Rodino Antitrust Improvements Act of 1976 (HSR Act).^12^

Box 1 **Relevant terminology**

**Table:** 

**Term**	**Meaning**
Holding period	the length of time an asset is owned or held by an investor before it is sold or otherwise disposed of
Spin-offs	when a parent company distributes shares to its shareholders to create a separate and fully independent company (see web resources)
Acquisition	when one company takes full control of another company
Vertical merger	a merger between companies at different stages of the same supply chain (e.g., supplier and manufacturer)
Horizontal merger	a merger between companies that are direct competitors in the same industry (see web resources)
Non-competition agreement	a contractual promise to avoid engaging in similar business for a stated time and stated area
Market efficiency	the idea that prices in a market reflect all available information
Nascent competitors	firms that are either new to the relevant market or entering the market soon
Commercialized competitors	firms that already sell products or services in a given market
*Pro rata* distribution of shares	when shares are divided among shareholders in proportion to the number of shares they already own
Consideration	something of value (such as provision of cash or a promise)
Open offer	an agreement guaranteeing customers access to a product or service, often at a set price or supply level

The Sherman Act (1890) serves as the foundation of US antitrust laws, prohibiting agreements that restrain trade and outlawing monopolization or attempts to monopolize. Violations carry severe penalties, including fines of up to $100 million for corporations and $1 million for individuals and potential imprisonment of up to 10 years^13^ The FTC Act builds on this foundation and prohibits “unfair methods of competition” and “unfair or deceptive acts or practices.”^14^ Importantly, any violation of the Sherman Act also constitutes a violation of the FTC Act. However, the FTC Act’s scope extends beyond the Sherman Act by addressing additional anti-competitive conduct that might not fall explicitly under the Sherman Act prohibitions (see web resources).

Recognizing the need to regulate mergers specifically, the Clayton Act was introduced to supplement the Sherman Act by targeting mergers and acquisitions that “may be substantially to lessen competition, or to tend to create a monopoly” (see web resources). While the Sherman Act deals with anti-competitive behaviors broadly, the Clayton Act focuses on mergers that could harm market efficiency and consumer welfare.

Regarding enforcement, the HSR Act^12^ introduced a pre-merger notification process that served as the gateway for FTC and DOJ antitrust review. The pre-merger notifications process requires companies above a certain threshold (based on the size of the transaction) to inform the FTC and DOJ prior to any mergers and requires them to undergo a waiting period before any finalization of the transaction, to ensure the regulators have a chance to assess anti-competitive effects. This act also grants private parties the ability to seek court orders to prevent anti-competitive practices. Together, these statutes create a comprehensive regulatory framework that ensures mergers and acquisitions do not undermine market efficiency or harm consumers. In 2021, the FTC also announced that it would return to requiring Prior Approvals and Prior Notice in merger orders (which had been rescinded in 1995), extending oversight to acquisitions that would be too small to trigger the Hart-Scott-Rodino reporting thresholds (see web resources).

### Policy on mergers

The regulatory framework for scrutinizing mergers has led antitrust authorities, particularly the FTC, to historically focus more heavily on horizontal mergers given their greater risk to competition. Formal guidelines were introduced to assess both horizontal and vertical mergers, aiming to standardize enforcement. The 2010 Horizontal Merger Guidelines provided a framework for assessing the competitive effects of horizontal mergers, emphasizing factors such as market concentration and potential harm to consumers (see web resources). The 2020 Vertical Merger Guidelines were introduced to address the unique challenges of vertical integrations, outlining how such mergers could lead to anti-competitive practices such as foreclosure or raising rivals’ costs (see web resources).

Under the leadership of former FTC Chairwoman Lina Khan, appointed by President Biden in 2021, there was a notable shift toward stricter enforcement of vertical mergers (see web resources). In January 2022, the FTC and DOJ jointly announced a comprehensive review of existing merger guidelines, seeking public input to modernize enforcement practices (see web resources). This initiative resulted in the release of updated merger guidelines on December 18, 2023, which lowered the thresholds for presumed anti-competitiveness and expanded the analytical framework to consider factors beyond price effects, such as eliminating potential entrants and reinforcing dominant market positions (see web resources). The *Illumina, Inc. vs. FTC*^15^ case (discussed below) serves as a prominent example of this enhanced scrutiny, where the FTC challenged a merger due to concerns over potential harm to nascent competitors.

The current FTC chairman, Andrew Ferguson, appointed on January 20, 2025, by President Trump, reaffirmed in February 2025 that the Commission intends to remain committed to enforcing the FTC and DOJ’s joint 2023 merger guidelines (see web resources). Additionally, the DOJ Antitrust Division announced the launch of a new Anticompetitive Regulations Task Force to begin the elimination of anti-competitive state and federal laws and regulations. This task force opened a 60-day public comment period for market participants to “identify unnecessary laws and regulations that raise the highest barriers to competition” (see web resources). Soon after, on April 9, 2025, the Trump administration issued Executive Order 14267, “Reducing Anti-Competitive Regulatory Barriers,” directing executive agency heads, in consultation with the attorney general and FTC chairman, to begin the process of eliminating anti-competitive regulations. The order states, "federal regulations should not predetermine new market entrants. Regulations that reduce competition, entrepreneurship, and innovation—as well as the benefits they create for American consumers—should be eliminated. This order commences the process for eliminating anti-competitive regulations to revitalize the American economy."^16^

The executive order created a blueprint for direct government action against anti-competitive rules by setting out timelines for the reporting of relevant information. For example, within 70 days of the order, the heads of all executive agencies were required to provide the DOJ and FTC with a list of all their regulations that imposed anti-competitive restrictions (such as the protection of monopolies). Also, within 90 days of receiving the lists, Ferguson, in consultation with the DOJ, agency heads, and the White House, is to present a consolidated list of these regulations to the Office of Management and Budget (OMB). The OMB director is then to consult with the FTC, agency heads, DOJ, and the White House to determine whether to implement the proposed rescissions and modifications as part of the administration’s deregulatory agenda. In September 2025, the FTC issued a recommendation that more than 125 regulations be scrapped or modified (see web resources).

Contrary to expectations of a shift toward a “pro-business” approach frequently construed as a relaxation of government scrutiny or reduced governmental interference with private business decisions, the Trump administration initially signaled a continuation of rigorous antitrust enforcement (see web resources). Ferguson emphasized the importance of stability in merger guidelines across administrations, rejecting the idea of wholesale rescission (see web resources). The decision to retain the 2023 guidelines suggested a continuation of strong merger enforcement, notwithstanding the April 2025 executive order that demonstrated an intent to promote competition through the elimination of perceived governmental obstacles.^16^ Abigail Slater, President Trump’s nominee to lead the DOJ’s Antitrust Division, also expressed agreement with the sentiment of maintaining consistency regarding the regulation of vertical mergers; however, Slater has demonstrated more openness to settling concerns with deals through merger remedies than the previous head of the division, Jonathan Kanter (from the Biden administration) (see web resources).

While the administration’s initial stance suggested continuity, the true impact of its approach to merger enforcement will become clearer over time as specific cases and policy decisions begin to unfold. Whereas the Biden administration took a more unified policy approach to promote business and combat anti-competitive practices (as outlined in Executive Order 14036), the Trump administration issued Executive Order 14337, rejecting that approach outright and rescinding Executive Order 14036. Nevertheless, the FTC has continued case-by-case efforts to challenge anti-competitive deals, and its proposed strategic plan for fiscal years 2026–2030 continues initiatives to address merger and non-merger anti-competitive conduct (see web resources).

Companies in the human genetics industry (and the broader biotech and pharmaceutical industries) should continue to expect scrutiny, particularly in cases involving high market concentration. The retention of the 2023 guidelines suggests that merger enforcement could remain rigorous, and companies should continue to make business decisions and structure deals with antitrust risk in mind.

### Policy on non-competes

Another relevant area that has seen a recent policy shift is the FTC’s position on the legality of non-competition agreements. In May 2024, the FTC issued the “Non-Compete Clause Rule,” effectively banning them nationwide (see web resources). The final rule, approved 3-2 by the five FTC commissioners, is based on its determination (substantiated by 30 “high-quality empirical studies from the last 21 years quantifying the harms caused by—not merely correlated with—noncompetes”) that such contractual provisions locking in employees are unfair methods of competition, violating Section 5 of the FTC Act (see web resources). A ban on non-competes is expected to increase innovation, increase the number of new startups, and increase worker earnings (see web resources). Then FTC Commissioner (now FTC Chairman) Andrew Ferguson voted against the final rule (see web resources) and, in a dissenting statement, conceded that such clauses have been viewed “with deep suspicion for centuries” but opened the door for courts to later invalidate the rule through application of a newly created “major questions doctrine.”^17^ The suggestion that the FTC lacked adequate authority to issue the non-competes rule was countered strongly—with citation to legal authority, extensive history of consistent enforcement activity by the FTC, and legal precedent—in a statement issued by then FTC Chairwoman Lina Khan, joined by Commissioners Rebecca Slaughter and Alvaro Bedoya (see web resources).

The status of this Non-Compete Clause Rule has been in question, as it was challenged almost immediately with three separate cases in three separate jurisdictions. The courts reached different conclusions on the rule, and the US District Court for the Northern District of Texas issued a ruling in August 2024 setting aside the FTC Non-Compete Clause Rule on the grounds that the FTC had exceeded its statutory authority and that the rule was overbroad in scope (thus making it “arbitrary and capricious”) (see web resources). While FTC appeals still appeared as pending as of July 2025 in the Fifth Circuit and Eleventh Circuit cases, in March 2025, the FTC (under the leadership of the Trump administration) paused the appeal for 120 days to review the FTC’s policy and position, effectively leaving in place the nationwide injunction issued by the Fifth Circuit that blocked the FTC’s enforcement of the non-competes rule (see web resources). The ability of courts to issue such nationwide injunctions, however, was greatly curtailed by the US Supreme Court in *Trump vs. Casa, Inc.*, decided on June 27, 2025, limiting injunctive relief only to the extent necessary and appropriate to provide the plaintiffs with complete relief (see web resources). As some law firms have explained, the impact of this Supreme Court decision could be far-reaching, as it is expected to cause reduced reliance on precedents, increased litigation (including but not limited to class actions), and further fragmentation of the regulatory landscape and corresponding compliance asymmetries (see web resources).

Notwithstanding the current legal uncertainty around the rule (which has been further compounded by the controversial firings of Commissioners Slaughter and Bedoya that are subject to separate litigation), it is apparent that the FTC Non-Compete Clause Rule will not take effect for the foreseeable future (see web resources). Before the abatement period in the cases expired, in September 2025, the FTC officially abandoned a categorical rule against non-compete agreements; however, enforcement against them has continued on a case-by-case basis (see web resources). Thus, it seems plausible that the use of non-competes to restrict geneticists from launching their own startups or working for other companies (with reasonable constraints on geographic scope and duration) could persist across the industry, along with non-compete clause alternatives (such as non-disclosure agreements and trade secret protections). Empirical research would be helpful to understand the extent to which such practices either affect the pace of innovation (as knowledge becomes concentrated in a few entities) or suppress entrepreneurship within the human genetics industry specifically.

### Policy on exclusive dealings

In addition to vertical and horizontal mergers and acquisitions and the use of non-compete clauses in employment contracts, exclusive dealings are a common practice to gain a competitive edge. Exclusive dealings refer to contractual provisions that require a party to do business exclusively with one partner (e.g., obligating a party to purchase equipment, products, or services from a single provider or to distribute or use only products from a single manufacturer). As a hypothetical example, exclusive dealings could mean that a company such as Myriad Genetics is contractually obligated to purchase DNA sequencing technologies from Illumina and prohibited from purchasing equipment from any other supplier in exchange for discount pricing or early access to new technology. Such arrangements are common in strategic partnerships (helping to promote both corporate brands) and often are lawful; however, exclusive dealings have the potential for misuse by powerful, established companies to block access to suppliers, retailers, or distributors in ways that make emerging new businesses struggle unfairly (see web resources).

Joint antitrust guidelines by the FTC and DOJ have long been available on the focused topic of licensing of intellectual property (IP), and these guidelines were updated in January 2017 (just before the transition between the Obama and Trump administrations) (see web resources). The updated guidance indicated that exclusivity typically would be evaluated under the “rule of reason” (i.e., “whether the restraint is likely to have anticompetitive effects and, if so, whether the restraint is reasonably necessary to achieve procompetitive benefits that outweigh those anticompetitive effects.”). The updated guidance further signaled that, on occasion, exclusivity (including instances of naked price-fixing or market division among horizontal competitors) would be considered anti-competitive *per se* (see web resources).

An aggressive antitrust approach continued throughout the Biden administration (see web resources). While there has been a wide range of abrupt changes in policy since January 2025 across the entire federal government, antitrust policy seems to be a rare area of relative stability thus far, including with continued litigation challenging exclusive contracts as violations of the Sherman Act (see web resources). Remarks given in May 2025 by FTC Commissioner Mark Meador supported the expectation that strong antitrust policy will continue at the FTC, as he explained (see web resources):

… innovation is too often treated … [as] a floating abstraction — a sweeping defense that excuses exclusionary conduct and avoids scrutiny by reframing anything on the path to dominance and power as progress. This focus on ends without considering means is not a defense of innovation – it is a veiled attempt to abandon the rule of law.

When the FTC has historically dedicated antitrust resources, it has done so in areas such as healthcare and biomed/biotech industries (see web resources). The human genetics industry would be safe assuming that this heightened scrutiny in healthcare and biomed/biotech will continue (see web resources).

## Illumina and Grail as a case study

In *Illumina,* the FTC first took action against the proposed merger of Illumina, a biotech firm, and Grail, a multi-cancer early detection (MCED) test developer.^15^ To block the merger, the FTC needed to demonstrate that Illumina’s acquisition of Grail would substantially harm competition by preventing nascent competitors from effectively competing in the market for MCED tests.^18^ The geographic market definition (important for determining the range where competition happens) was uncontested and defined as the United States, while the product market definition (important for determining what products or services are competing with each other) was a key point of dispute. The FTC argued that MCED tests constituted a distinct market separate from other cancer screening tests, as they use next-generation sequencing (NGS) technology to detect multiple cancers from a single blood sample. In contrast, traditional screening methods typically detected only one type of cancer at a time and relied on different diagnostic processes. To address anti-competitive concerns, Illumina proposed an Open Offer, a supply agreement guaranteeing oncology customers access to its NGS platform.

The administrative law judge (ALJ) who heard the initial arguments dismissed the FTC’s challenge in September 2022,^19^ ruling that the agency had failed to prove substantial harm to competition. However, the FTC’s staff prosecutors appealed the ALJ’s ruling to the full FTC Commission for a *de novo* review. In April 2023, the FTC commissioners overturned the ALJ’s ruling on appeal and ordered Illumina to divest Grail, citing evidence that the Open Offer provided by Illumina would be too difficult to enforce and monitor and would not sufficiently eliminate competitive risk. The FTC further asserted that Illumina had both the incentive and ability to raise rivals’ costs or restrict access to its sequencing technology, harming competition in the MCED test market.^20^

**Figure 1:**
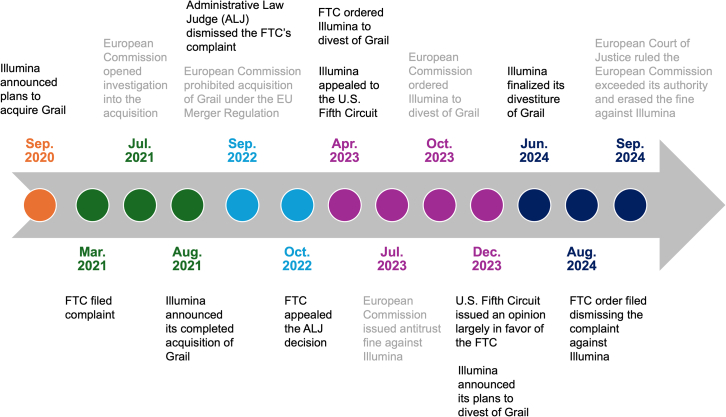
Overview of Illumina/Grail case timeline Gray text is used to distinguish events occurring in the European Union from those occurring in the United States, which are displayed in black.

Following the ultimate ruling by the FTC, Illumina then appealed to the US Court of Appeals for the Fifth Circuit. In December 2023, the court largely upheld the FTC’s ruling but remanded the case for reconsideration of how the Open Offer should be evaluated.^18^ Facing legal uncertainty, Illumina voluntarily announced it would divest Grail rather than pursue further litigation (see web resources). By June 2024, the divestiture process was completed, and the case was formally closed shortly after. Figure 1 displays the timeline for the case.

While the divestment was a business decision by Illumina and not based strictly on a court order, the outcome of this case underscores the heightened scrutiny of vertical mergers in the biotechnology sector, signaling potential challenges for future acquisitions. Companies considering vertical mergers need to specifically consider potential nascent competitors, as opposed to just similar commercialized competitors. The scrutiny of Illumina’s proposed Open Offer reveals that those attempting to merge should have a clear plan for remedies and demonstrate that the merger would not result in anti-competitive effects. Failing to address antitrust concerns early could trigger immediate regulatory scrutiny, especially given the FTC’s reliance on information provided through Prior Approval and Prior Notice Provisions in merger orders. Having a well-developed plan, with anticipation of the issues likely to be flagged, can prevent issues that can lead to heightened scrutiny.

## Anticipated impacts: A reduction of vertical mergers

The continuation of this modern, more aggressive approach could reduce the number of proposed vertical mergers, especially in the biotechnology industry. This could require adaptation and the development of new strategies to develop these technologies through other means.

### Spin-offs

The biotechnology field has historically leaned on spin-offs.^21^ This was how Grail initially became independent in 2016, as it was spun off from Illumina to focus specifically on MCED test development. Traditionally, spin-offs required *pro rata* distribution of shares, no consideration paid by shareholders, a valid business purpose, and a holding period of at least one year.^22^ However, recent proposed regulations issued by the IRS and the Treasury Department in January 2025 introduced stricter limits and new multi-year reporting obligations for corporate separations.^23^ These changes could significantly impact spin-off strategies in biotechnology, potentially leading to companies refraining from spinning off and focusing instead on the in-house development of new technologies. Larger companies could view spin-offs as a bigger risk that could result in them being unable to re-acquire their assets.

### Startup struggles

In lieu of spinning off, startups will likely struggle to access the capital they need to continue their specialized research. More stringent analysis of mergers could, in turn, make it more difficult for startups to survive. When a startup first comes into existence, it usually has substantially negative cash flow and relies on investors interested in developing new innovative ideas.^24^ This development of niche technologies could be made more difficult for startups without investors, who could hesitate to invest if they do not see a straightforward and quick return on their investment. Typically, larger firms would seek the fastest return on investment possible by acquiring the emerging technology developed by the startup and supported by the investors who provided the funds and support to initially develop this new, niche technology. Through this acquisition, larger firms would benefit by increasing their IP portfolios with emerging technology while reducing risk with somewhat proven technology. This *quid pro quo* fostered a cycle of incentives that rewarded innovation and continued to push the industry further over time.

However, increased scrutiny of vertical mergers and acquisitions could leave new companies struggling to find a way to enter the market sustainably. With limited financing available for new projects, there is a risk that less immediately profitable technologies could be overlooked in favor of shorter-term gains, as profit-oriented investors could be less inclined to invest in startups.

### A move toward in-house development

A move toward more extensive in-house research and development could become the norm, as having the labs and IP under the parent company would shield the company from the need to merge. While in-house R&D comes with security against mergers, it also comes with the downside of paying the initial research and development costs. The development costs of a new drug are an average of $879.3 million, and the development can range from 10 to 15 years, with no guarantee of success.^25^ This uncertainty regarding potential return on investments could result in stagnation in the field, where firms are more hesitant to invest resources into less-researched areas. While the companies that initially developed in-house will continue to do so, maintaining the level of innovation typically brought by startups could become more difficult, as securing initial funding for new ideas will be harder without the feasibility of spin-offs or creating startups. Ultimately, if larger companies become more reluctant to acquire startups or spin-off, researchers with innovative ideas may face higher barriers to securing funding, making it more challenging to bring new developments to market.

## Concluding remarks

Having a basic understanding of the statutory authority, policy positions, and enforcement activities of the FTC could help geneticists anticipate opportunities and obstacles in taking their scientific innovations from the laboratory to the marketplace.

While it is impossible to predict policy and enforcement decisions with certainty in a deregulatory era and while there have been dramatic shifts in federal policy since January 2025, the FTC merger guidelines appear to be a rare exemplar of stability. Thus, it is possible to identify certain business actions that will likely prompt closer regulatory scrutiny by the FTC. It is reasonable to expect that mergers (in and out of the human genetics industry) will receive increased scrutiny and that there will be a broader focus on nascent competition and more intense attention given to roll-ups and early-stage acquisitions. Continued attention on exclusivity is also likely to continue. Conversely, it seems rather unlikely that the FTC will take major steps to curtail the use of non-competes. Other areas having “pro-business” implications and raising concerns about consumer protection and market competitiveness (such as the FTC’s authority to require substantiation of advertising claims with scientific evidence, which is currently subject to litigation) should be monitored as well.

While we have outlined a few distinct types of anti-competitive practices that attract regulatory scrutiny (namely, aggressive mergers and acquisitions, non-compete clauses, and exclusive dealings), such practices are rarely taken in isolation. Indeed, as prominent legal scholars have wisely illuminated,^26^ the interactive and cumulative effects of business practices can be significant. Accordingly, regulatory scrutiny of antitrust practices in such a way is problematic, overlooking both probabilistic harms (in which multiple acts collectively present serious anti-competitive risks even if the acts individually carry only a small chance of harm) and synergistic harms (in which harmful anti-competitive effects are caused by a combination of activities that are individually legal).^26^ At least for the foreseeable future, however, it seems unlikely that either the FTC or the courts will adopt a holistic anti-competition framework.

The more aggressive enforcement of vertical mergers, exemplified by the Illumina/Grail case, highlights the need for a deeper understanding of their impact on consumers. This shift in regulatory scrutiny suggests a departure from traditional pathways of technological development, such as spin-offs and startups. As a result, firms in the US might need to reconsider their strategies for innovation and growth, staying informed on evolving anti-competition policies. With greater uncertainty in the regulatory landscape, companies might shift toward in-house research and development, joint development projects with other large firms, or IP acquisitions with smaller companies rather than relying on spin-offs and startups. Additionally, firms should stay abreast of developments in antitrust policies elsewhere in the world when deciding where, with whom, and how to do business.

This shift in approach in US policy raises important questions about the balance between fostering innovation and maintaining fair competition. Research into the ethical, legal, and social implications can provide data for regulators and legislators to determine what actions are needed to ensure fair competition. As the landscape of vertical mergers continues to evolve, both companies and regulatory bodies need to adapt their strategies and policies accordingly. This will help balance encouraging technological advancements and preserving a competitive market environment that ultimately benefits consumers.

## Data and code availability

This study did not generate/analyze datasets/code.

## Acknowledgments

This work was supported in part by grant no. R01HG011051 from the National Human Genome Research Institute (NHGRI) and in part by grant no. R21EB035474, awarded by the National Institutes of Health Office of the Director (NIH OD) and the National Institute of Biomedical Imaging and Bioengineering (NIBIB). The content of this perspective is the authors’ responsibility and might not represent the views of the authors’ current or former funding sources, employers, clients, or any other person or entity.

## Author contributions

Investigation, A.I.R., N.A.R., N.R., and J.K.W.; writing – original draft, A.I.R., N.A.R., N.R., and J.K.W.; writing – review & editing, A.I.R., N.A.R., and J.K.W.; conceptualization, J.K.W.; supervision, J.K.W.; funding acquisition, J.K.W.; project administration, J.K.W.

## Declaration of interests

The authors declare no competing interests.

## Declaration of generative AI and AI-assisted technologies in the writing process

The authors have nothing to declare.

## Web resources

Servick, K. 23andMe bankruptcy filing leaves future of genetic data trove uncertain. Science. Mar. 24, 2025, https://www.science.org/content/article/23andme-bankruptcy-filing-leaves-future-genetic-data-trove-uncertain (Last accessed Dec. 2, 2025)
Spector-Bagdady, K. 23andMe is potentially selling more than just genetic data – the personal survey info it collected is just as much a privacy problem. The Conversation, Apr. 2, 2025, https://theconversation.com/23andme-is-potentially-selling-more-than-just-genetic-data-the-personal-survey-info-it-collected-is-just-as-much-a-privacy-problem-253220 (Last accessed Dec. 2, 2025)
FTC Press Release, “Federal Trade Commission Chairman Andrew N. Ferguson Issues Letter on 23andMe Bankruptcy Impact to Consumers,” Mar. 31, 2025, https://www.ftc.gov/news-events/news/press-releases/2025/03/federal-trade-commission-chairman-andrew-n-ferguson-issues-letter-23andme-bankruptcy-impact (Last accessed Dec. 2, 2025)
Press Release U.S. Senator Mark Warner, “Warner, Young Push DOJ, FTC to Use Every Available Resource to Protect Americans' Data Amid 23andMe Bankruptcy Proceedings,” April 29, 2025, https://www.warner.senate.gov/public/index.cfm/2025/4/warner-young-push-doj-ftc-to-use-every-available-resource-to-protect-americans-data-amid-23andme-bankruptcy-proceedings (Last accessed Dec. 2, 2025)
E.g., McArthur, D. “Guest post by Daniel Vorhaus and Lawrence Moore: what happens when a personal genomics company goes bankrupt?” Wired, Sep. 4, 2009, https://www.wired.com/2009/09/guest-post-by-daniel-vorhaus-and-lawrence-moore-what-happens-when-a-personal-genomics-company-goes-bankrupt/ (Last accessed Dec. 2, 2025)
See, e.g., Greely, H. “Amgen Buys DeCODE – Reflections Backwards, Forwards, and on DTC Genomics,” Stanford Law and Biosciences Blog, Dec. 13, 2012, https://law.stanford.edu/2012/12/13/lawandbiosciences-2012-12-13-amgen-buys-decode-reflections-backwards-forwards-and-on-dtc-genomics/ (Last accessed Dec. 2, 2025)
Roche announces $5.7 billion hostile takeover bid for Illumina. Genomeweb, Jan. 25, 2012, https://www.genomeweb.com/clinical-sequencing/roche-announces-57-billion-hostile-takeover-bid-illumina#.YrC9UOzMKWA (Last accessed Dec. 2, 2025)
Roche won’t extend offer for Illumina as shareholders re-elect Flatley, others to board. Genomeweb, Apr. 18, 2012, https://www.genomeweb.com/sequencing/roche-wont-extend-offer-illumina-shareholders-re-elect-flatley-others-board#.YrDC8-zMKWA (Last accessed Dec. 2, 2025)
Petrone, J. FTC’s second request to Roche could lead to NimbleGen divestiture, analysts say. Genomeweb, Mar. 21, 2012, https://www.genomeweb.com/arrays/ftcs-second-request-roche-could-lead-nimblegen-divestiture-analysts-say#.YoaHnajMK3A (Last accessed Dec. 2, 2025)
Friese, S. Concerns over Chinese genomics bid, Dec. 4, 2012, Politico, https://www.politico.com/story/2012/12/concerns-arise-in-chinese-bid-for-genomics-firm-084516 (Last accessed Dec. 2, 2025)
Zhang, H. Illumina, BGI, Complete Genomics Lobbying Congress on Bills Targeting BGI Group, Affiliates, Apr. 23, 2024, Genome Web, https://www.genomeweb.com/sequencing/illumina-bgi-complete-genomics-lobbying-congress-bills-targeting-bgi-group-affiliates (Last accessed Dec. 2, 2025)
Service, R. F. Bill targeting Chinese biotechs worries U.S. researchers, Science, Sep. 13, 2024, https://www.science.org/content/article/bill-targeting-chinese-biotechs-worries-u-s-researchers (Last accessed Dec. 2, 2025)
Spin-off, The Law Dictionary (featuring Black’s Law Dictionary, 2d ed.), https://thelawdictionary.org/spin-off/ (Last accessed Dec. 2, 2025)
Horizontal Merger, The Law Dictionary (featuring Black’s Law Dictionary, 2d ed.), https://thelawdictionary.org/horizontal-merger/ (Last accessed Dec. 2, 2025)
FTC, Guide to Antritrust Laws, https://www.ftc.gov/advice-guidance/competition-guidance/guide-antitrust-laws/antitrust-laws (Last accessed Dec. 2, 2025)
Fed. Trade Comm’n, Statement of the Commission on Use of Prior Approval Provisions in Merger Orders (Oct. 25, 2021), https://www.ftc.gov/system/files/documents/public_statements/1597894/p859900priorapprovalstatement.pdf (Last accessed Dec. 2, 2025)
U.S. Dep’t of Just. & Fed. Trade Comm’n, Horizontal Merger Guidelines (Aug. 19, 2010), https://www.justice.gov/atr/horizontal-merger-guidelines-08192010 (Last accessed Dec. 2, 2025)
U.S. Dep’t of Just. & Fed. Trade Comm’n, Vertical Merger Guidelines (Jun. 30, 2020) (withdrawn 2021), https://www.ftc.gov/legal-library/browse/cases-proceedings/public-statements/vertical-merger-guidelines. (Last accessed Dec. 2, 2025)
Press Release, Fed. Trade Comm’n, Statement of FTC Chair Lina M. Khan and Antitrust Division Acting Assistant Attorney General Richard Powers on Competition (Jul. 9, 2021), https://www.ftc.gov/news-events/news/press-releases/2021/07/statement-ftc-chair-lina-m-khan-antitrust-division-acting-assistant-attorney-general-richard-powers (Last accessed Dec. 2, 2025)
Statement of Chair Lina M. Khan Regarding Request for Information on Merger Enforcement, Fed. Trade Comm’n (Jan. 18, 2022), https://www.ftc.gov/legal-library/browse/cases-proceedings/public-statements/statement-chair-lina-m-khan-regarding-request-information-merger-enforcement (Last accessed Dec. 2, 2025)
U.S. Dep’t of Just. & Fed. Trade Comm’n, 2023 Merger Guidelines (Dec. 18, 2023), https://www.ftc.gov/system/files/ftc_gov/pdf/p859910mergerguidelines2023.pdf (Last accessed Dec. 2, 2025)
Memorandum from Andrew Ferguson, Chairman, Fed. Trade Comm’n, on Merger Guidelines (Feb. 2025), https://www.ftc.gov/system/files/ftc_gov/pdf/ferguson-memo-re-merger-guidelines.pdf (Last accessed Dec. 2, 2025)
Bigelow, B. *DOJ Task Force Seeks to Root Out Anticompetitive Regulations in Transportation Industry*, JD Supra (last visited Apr. 30, 2025*),* https://www.jdsupra.com/legalnews/doj-task-force-seeks-to-root-out-4705160/ (Last accessed Dec. 2, 2025)
FTC, *FTC Recommends Anticompetitive Regulations for Deletion or Revision* (Sept. 17, 2025), https://www.ftc.gov/news-events/news/press-releases/2025/09/ftc-recommends-anticompetitive-regulations-deletion-or-revision (Last accessed Dec. 2, 2025)
Jacobsen, R. A., Jr., Dubrow, J. B., and Grosberg, J. (2024, December 4). *What a Second Trump Term Means for Antitrust Enforcement.* National Law Review. Retrieved from https://www.natlawreview.com/article/what-second-trump-term-means-antitrust-enforcement (Last accessed Dec. 2, 2025)
Memorandum from Andrew Ferguson, Chairman, Fed. Trade Comm’n, on Merger Guidelines (Feb. 2025), https://www.ftc.gov/system/files/ftc_gov/pdf/ferguson-memo-re-merger-guidelines.pdf (Last accessed Dec. 2, 2025)
Slater, A. Responses to Written Questions of Senator Maizie K. Hirono for Hearing on “Nominations” (Feb. 17, 2025), https://www.judiciary.senate.gov/imo/media/doc/2025-02-12_-_qfr_responses_-_slater.pdf (Last accessed Dec. 2, 2025)
Hearing of the U.S. Senate Judiciary Committee, “Nominations” (Feb. 12, 2025), https://www.judiciary.senate.gov/committee-activity/hearings/02/12/2025/nominations (Last accessed Dec. 2, 2025)
FTC, *FTC Invites Public Comment on Draft Strategic Plan*, (Sept. 26, 2025), https://www.ftc.gov/news-events/news/press-releases/2025/09/ftc-invites-public-comment-draft-strategic-plan (Last accessed Dec. 2, 2025)
Press Release: FTC Announces Rule Banning Noncompetes, April 23, 2024, https://www.ftc.gov/news-events/news/press-releases/2024/04/ftc-announces-rule-banning-noncompetes (subsequently published in FTC, Non-Compete Clause Rule, 89 FR 38342 (May 7, 2024)) (Last accessed Dec. 2, 2025)
Statement of Chair Lina M. Khan Joined by Commissioner Rebecca Kelly Slaughter and Commissioner Alvaro M. Bedoya Regarding the Non-Compete Clause Final Rule, Dec. 31, 2024 https://www.ftc.gov/legal-library/browse/cases-proceedings/public-statements/statement-chair-lina-m-khan-joined-commissioner-rebecca-kelly-slaughter-commissioner-alvaro-m-bedoya-regarding-non-compete-clause-final-rule (Quotation at p20) (Last accessed Dec. 2, 2025)
Ferguson, A. N., Chairman, Oral Statement of Commissioner Andrew N. Ferguson In the Matter of the Non-Compete Clause Rule, Apr. 23, 2024, Available at https://www.ftc.gov/system/files/ftc_gov/pdf/ferguson-oral-statement-noncompete.pdf (Last accessed Dec. 2, 2025)
See, e.g., Clark, B. S. *One Year Later: What is the Status of the FTC’s Non-Compete Rule?* May 1, 2025, Shields Legal, https://shieldslegal.com/one-year-later-what-is-the-status-of-the-ftcs-non-compete-rule/ (discussing Ryan, LLC v. Federal Trade Commission, No. 3:24-cv-00986 [N.D. Tex. Aug. 20, 2024]; ATS Tree Services, LLC v. Federal Trade Commission, No. 2:24-cv-01743-KBH [E.D. Pa. July 23, 2024]; and Properties of the Villages, Inc. v. Federal Trade Commission, No. 5:24-cv-316 [M.D. Fla. Aug. 15, 2024]) (Last accessed Dec. 2, 2025)
See, e.g., Schwartz, D. E., Rider-Longmaid, P., and Rancour, J. M. “FTC Noncompete Rule is Set Aside, But Appeal is Expected and States May Act,” Harvard Law School Forum on Corporate Governance, Sept. 5, 2024, https://corpgov.law.harvard.edu/2024/09/05/ftc-noncompete-rule-is-set-aside-but-appeal-is-expected-and-states-may-act/ (Last accessed Dec. 2, 2025)
Trump, President of the United States, et al. v. Casa, Inc. et al., No. 24A884, Jun. 27, 2025, https://www.supremecourt.gov/opinions/24pdf/24a884_8n59.pdf (Last accessed Dec. 2, 2025)
E.g., Sidley Austin, Supreme Court Substantially Limits Universal Injunctions (Trump v. CASA): Implications for Litigation Against the Government, Jul. 1, 2025, https://www.sidley.com/en/insights/newsupdates/2025/07/supreme-court-substantially-limits-universal-injunctions#:∼:text=On%20June%2027%2C%202025%2C%20the,the%20plaintiffs%20before%20the%20court (Last accessed Dec. 2, 2025)
Reuters, “Judge rules Trump’s firing of FTC commissioner was illegal” Jul. 17, 2025, https://www.cnn.com/2025/07/17/business/ftc-firings-ruling-trump (Last accessed Dec. 2, 2025)
FTC, *Federal Trade Commission Files to Accede to Vacatur of Non-Compete Clause Rule*, Sept. 5, 2025, https://www.ftc.gov/news-events/news/press-releases/2025/09/federal-trade-commission-files-accede-vacatur-non-compete-clause-rule (Last accessed Dec. 2, 2025)
See, e.g., FTC, Exclusive Dealing or Requirements Contracts, n.d. https://www.ftc.gov/advice-guidance/competition-guidance/guide-antitrust-laws/dealings-supply-chain/exclusive-dealing-or-requirements-contracts#:∼:text=In%20simple%20terms%2C%20an%20exclusive,inputs%20from%20a%20different%20supplier (Last accessed Dec. 2, 2025)
U.S. DOJ and FTC. Antitrust Guidelines for the Licensing of Intellectual Property. Apr. 6, 1995, https://www.ftc.gov/sites/default/files/attachments/competition-policy-guidance/0558.pdf (Last accessed Dec. 2, 2025)
U.S. DOJ and FTC. Antitrust Guidelines for the Licensing of Intellectual Property. Jan. 12, 2017, https://www.justice.gov/atr/IPguidelines/dl (Last accessed Dec. 2, 2025)
Sibarium, M. L., Polidora, R. A., Navikas, D. A., Kinser, S. O., and Warley, M. A. “Client briefing: Biden’s blueprint for aggressive antitrust enforcement,” Pillsbury, Nov. 15, 2021, https://www.pillsburylaw.com/en/news-and-insights/biden-antitrust-enforcement.html (Last accessed Dec. 2, 2025)
Owings, T. M., Ohlhausen, M. Ferris, J. P., Bank, J. C., and Wentross, E. Enforcement Priorities of President Trumps DOJ and FTC Begin to Take Shape, Wilson Sonsini, Feb. 26, 2025, https://www.wsgr.com/en/insights/enforcement-priorities-of-president-trumps-doj-and-ftc-begin-to-take-shape.html (Last accessed Dec. 2, 2025)
“Meet the New Boss, Not So Different From the Old Boss? Antitrust in the Trump Era,” WilmerHale Client Alert, Jun. 2, 2025, https://www.wilmerhale.com/en/insights/client-alerts/20250602-meet-the-new-boss-not-so-different-from-the-old-boss-antitrust-in-the-trump-era (Last accessed Dec. 2, 2025)
Meader, M. R. “Antitrust Myth Busting Mark R. Meador Commissioner, U.S. Federal Trade Commission Remarks at the Second Annual Antitrust Conference at the George Washington University presented by the GW Competition & Innovation Lab” Available at https://www.ftc.gov/system/files/ftc_gov/pdf/meador-antitrust-myth-busting-remarks-5.5.25.pdf at 2 (Last accessed Dec. 2, 2025)
FTC, Competition Counts, https://www.ftc.gov/advice-guidance/competition-guidance/competition-counts (stating “Through its Bureaus of Competition and Economics, the FTC puts its antitrust resources to work, especially where consumer interest and spending are high: in matters affecting energy, health care, food, pharmaceuticals, professional services, computer technology and databases, medical devices, and funeral services”) (Last accessed Dec. 2, 2025)
See, e.g., Whittaker, B., Lang, G., and Medina, M. “Antitrust priorities in the second Trump administration” Nixon Peabody, May 5, 2025, https://www.nixonpeabody.com/insights/alerts/2025/05/05/antitrust-priorities-second-trump-administration (Last accessed Dec. 2, 2025)
Press Release, Illumina, Inc., Illumina to Divest GRAIL (Dec. 17, 2023), https://investor.illumina.com/news/press-release-details/2023/Illumina-Announces-Decision-to-Divest-GRAIL/default.aspx (Last accessed Dec. 2, 2025)
